# Pathogenic LRRK2 Mutations Do Not Alter Gene Expression in Cell Model Systems or Human Brain Tissue

**DOI:** 10.1371/journal.pone.0022489

**Published:** 2011-07-22

**Authors:** Michael J. Devine, Alice Kaganovich, Mina Ryten, Adamantios Mamais, Daniah Trabzuni, Claudia Manzoni, Philip McGoldrick, Diane Chan, Allissa Dillman, Julia Zerle, Susannah Horan, Jan-Willem Taanman, John Hardy, Jose-Felix Marti-Masso, Daniel Healey, Anthony H. Schapira, Benjamin Wolozin, Rina Bandopadhyay, Mark R. Cookson, Marcel P. van der Brug, Patrick A. Lewis

**Affiliations:** 1 Department of Molecular Neuroscience, UCL Institute of Neurology, London, United Kingdom; 2 Laboratory of Neurogenetics, National Institute on Aging, National Institutes of Health, Bethesda, Maryland, United States of America; 3 Rita Lila Weston Institute and Queen Square Brain Bank, UCL Institute of Neurology, London, United Kingdom; 4 MRC Centre for Neuromuscular Disease, UCL Institute of Neurology, London, United Kingdom; 5 Department of Pharmacology, Boston University School of Medicine, Boston, Massachusetts, United States of America; 6 Department of Clinical Neuroscience, UCL Institute of Neurology, London, United Kingdom; 7 Hospital Donastia, San Sebastian, Spain; 8 The Scripps Research Institute, Jupiter, Florida, United States of America; Newcastle University, United Kingdom

## Abstract

Point mutations in LRRK2 cause autosomal dominant Parkinson's disease. Despite extensive efforts to determine the mechanism of cell death in patients with LRRK2 mutations, the aetiology of LRRK2 PD is not well understood. To examine possible alterations in gene expression linked to the presence of LRRK2 mutations, we carried out a case versus control analysis of global gene expression in three systems: fibroblasts isolated from LRRK2 mutation carriers and healthy, non-mutation carrying controls; brain tissue from G2019S mutation carriers and controls; and HEK293 inducible LRRK2 wild type and mutant cell lines. No significant alteration in gene expression was found in these systems following correction for multiple testing. These data suggest that any alterations in basal gene expression in fibroblasts or cell lines containing mutations in LRRK2 are likely to be quantitatively small. This work suggests that LRRK2 is unlikely to play a direct role in modulation of gene expression, although it remains possible that this protein can influence mRNA expression under pathogenic cicumstances.

## Introduction

Mutations in Leucine Rich Repeat Kinase 2 (LRRK2) are the most common Mendelian genetic cause of Parkinson's disease (PD) currently identified [1]. Additionally, variation at this locus has recently been implicated as a risk factor for sporadic PD in two genome wide association studies [2], [3]. Although the function of LRRK2 is unknown, the presence of a kinase domain, a GTPase domain and protein/protein interaction domains within its open reading frame has led to suggestions that it could act as a signaling node within cells, with the protein/protein interaction regions functioning as scaffolding areas to recruit binding partners and substrates to an active complex [4], [5]. LRRK2 has been implicated in a number of signaling pathways including ERK signaling and the mTOR pathway [4], [6], [7]. It is likely that if LRRK2 does act in this manner, then alterations in the activity of this protein would result in changes in signaling pathways leading to downstream shifts in gene expression.

In addition, a recent study has suggested that pathogenic forms of LRRK2 bind directly to the miRNA processing enzyme Argonaute and thus influence mRNA levels in a *Drosophila* model of LRRK2 disease [8]. Because mammalian miRNAs potently reduce mRNA levels [9], the prediction from the *Drosophila* data would be that steady state mRNA levels of miRNA targets are altered in the presence of mutant LRRK2. To test whether mutations in LRRK2 cause an alteration in basal gene expression we have examined the impact of mutations in three contexts: fibroblast cells cultured from PD patients carrying mutations in LRRK2 (including the R1441G, Y1699C and G2019S mutations), brain tissue from 5 patients carrying the G2019S mutation compared to idiopathic PD and control brain tissue [10], and HEK293T cells stably transfected with a plasmid allowing inducible expression of LRRK2 with or without the R1441C mutation. We have compared global gene expression in these systems, searching for differences between wild type and mutant conditions.

## Materials and Methods

### Fibroblast culture

Fibroblast cultures were isolated from PD patients with LRRK2 mutations and healthy controls (summarised in table 1) following approval of the study by the ethics board of the Royal Free Hospital, London, UK and informed written consent from the individuals concerned. Skin punch biopsies (forearm) were cultured in 5 cm^2^ sterile Petri dishes at 37°C in 5% CO_2_ in 2 ml DMEM (supplemented with 10% Fetal Bovine Serum and 1% Penicillin/Streptomycin solution) until fibroblasts were seen to migrate from the skin explants [11]. Media volume was increased by 0.5 ml every 2 days. When plates were confluent, fibroblasts were lifted from dishes by trypsinising with TrypLE (Invitrogen) and transferred to 10 cm^2^ dishes for culturing. Samples were designated anonymous identifiers following collection. For collection of RNA, cells were plated in 10 cm plates at equivalent passage number (*n* = 7) and at equal density. Following 24 hours growth, RNA was extracted using the RNeasy kit (Qiagen) and RNA quality ascertained prior to microarray analysis using RIN analysis (Agilent). Western blot analysis of endogenous LRRK2 expression was carried out by immunoblotting following BCA assay (Pierce, as per manufacturers instructions), with 10 µg of lysate loaded onto 4–12% Bis Tris gels (Invitrogen) following denaturation in 4x sample buffer (Invitrogen) supplemented with 5% β-Mercaptoethanol (Sigma). Proteins were transferred to PVDF membrane (Millipore) and membranes blocked with 5% milk solution in Phosphate buffered saline for 1 hour prior to probing with primary (90minutes) and secondary (60minutes) antibodies. For LRRK2, rabbit monoclonal MJFF2 (Epitomics – see http://www.pdonlineresearch.org/discussions/lrrk2-antibodies for characterisation of these antibodies) was used at a 1∶1000 dilution, for β Actin mouse monoclonal AC-74 (Sigma) was used at a 1∶5000 dilution. HRP secondary antibodies were used at a dilution of 1∶2000 and 1∶10000 for LRRK2 (rabbit) and β Actin (mouse) respectively. Membranes were washed three times in PBS supplemented with 1% tween, and exposed to Supersignal ECL substrate (Pierce). Membranes were exposed to Kodak biomax film and developed on a Fujifilm developer as per manufacturers instructions.

**Table 1:** Patient and control fibroblast samples used in this study.

Mutation	LRRK2 Domain	*N*
R1441G	ROC	2
Y1699C	COR	1
G2019S	Kinase	8
Controls	N/A	11

### HEK293t LRRK2 expression

Flp-In-TRex-LRRK2 293 inducible cell lines [12] were grown in DMEM with 10%FBS, Pen/Strep, Blasticidin (15 ug/ml final conc.), Hygromycin (100 ug/ml final conc.). To induce LRRK2 protein expression, Doxycyclin (1 ug/ml final conc) was added to growth medium for 24–48 hours. LRRK2 expression was detected using mouse V5 Ab (Invitrogen) against V5 tag for Western blot and/or ICC. For Western Blotting and Illumina assay, inducible LRRK2 cells were seeded at 0.9×10^6^ cells/well in 6-well plates. Cells were induced with Doxycyclin and harvested at different time points with either 0.5 ml Trizol for RNA extraction or PBS for immunoblot analysis as described above. Expressed LRRK2 was demonstrated to possess kinase activity as previously described [13], [14].

### Brain samples

Occipital cortex samples were obtained from the Queen Square Brain bank (four G2019S samples and idiopathic PD control samples) and Sun Health brain bank (one G2019S sample and 36 neuropathologically examined control samples) [15]. Details of the brain samples used in this study are summarised in table 2. Samples from both sites had fully informed consent for retrieval and were authorized for ethically approved scientific investigation (Research Ethics Committee number 10/H0716/3).

**Table 2:** Mutant and idiopathic PD occipital cortex samples used in this study.

Case	Age at death	Sex	Pathology	Post mortem delay
G2019S 1	84	F	Limbic	32.2
G2019S 2	80	F	Limbic	44.4
G2019S 3	81	F	Limbic	15
G2019S 4	72	F	Limbic	24.55
G2019S 5	85	M	Limbic	2
IPD 1	78	F	Neocortical	60.3
IPD 2	73	F	Neocortical	29
IPD 3	84	F	Limbic	30
IPD 4	77	M	Limbic	41
IPD 5	85	M	limbic	49

### Human oligonucleotide arrays

Illumina HumanWG-6 and HumanHT-12, and Affymetrix Exon 1.0 ST Arrays were used according to manufacturer's instructions as previously described [16]. Briefly, 500 ng of total RNA was processed for each sample and quality control using RNA integrity number (RIN) performed prior to analysis [17]. Expression data was subjected to quantile normalization and differential expression values calculated on sets of biological replicates. The threshold for significance for individual genes was set at +/−1.5 fold expression between conditions and P<0.05. Cluster analyses were derived using the Illumina Beadstudio software suite. Expression data from this study are MIAME compliant and have been deposited in the GEO database (accession number GSE25580).

## Results

**Figure 1:**
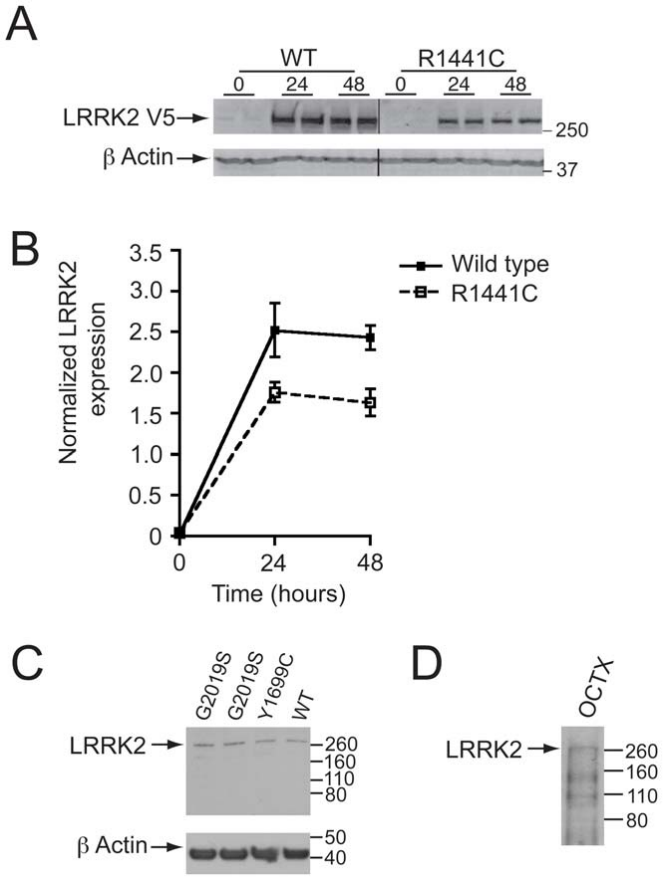
LRRK2 expression in inducible HEK cell lines and primary fibroblast cultures. (A) Induction of LRRK2 expression following exposure of cells to Doxycyclin as measured by immunoblotting for V5 epitope (B) Normalised protein expression of wildtype and R1441C plotted over time (C) Western blot analysis of endogenous LRRK2 expression in representative mutant and wild type fibroblast lines (D) Western blot analysis of LRRK2 in human occipital cortex.

We compared gene expression in three systems: an inducible HEK cell model, patient fibroblasts and patient brain samples for wild type and mutant LRRK2. To confirm expression of LRRK2 in these systems, we carried out western blot analysis using anti-V5 and anti-LRRK2 antibodies for the HEK cells and fibroblasts/brains samples respectively (Figure 1). In the HEK cells, basal expression of LRRK2 was low but was increased after induction within 24–48 hours. Expression levels were similar in both WT and R1441C lines (Figure 1A and 1B). We also confirmed expression of WT and mutant (G2019S and Y1699C) LRRK2 in fibroblast samples (Figure 1C), which again showed similar levels of expression. Finally, we assessed LRRK2 protein levels in occipital cortex (Figure 1D), demonstrating expression of full length LRRK2.

**Figure 2:**
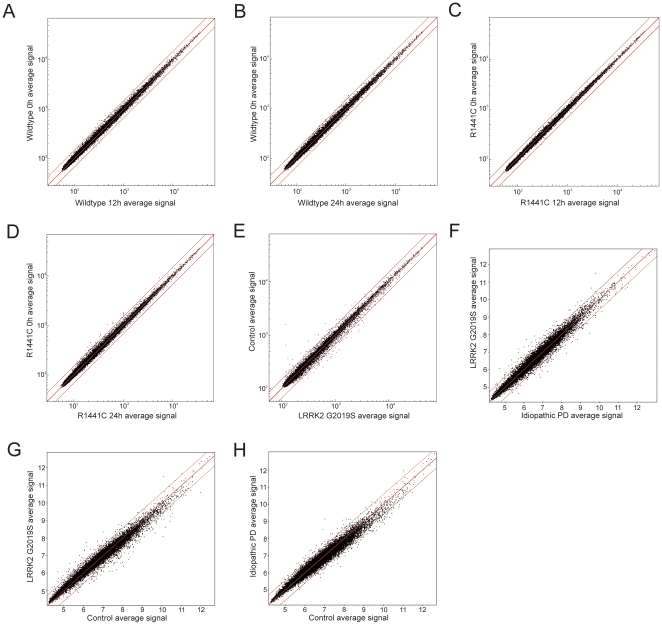
Gene expression scatterplot analysis. (A) Uninduced expression state of HEK cells stably transfected with a construct expressing WT LRRK2 under the control of an inducible promoter (y axis) vs. cells induced for 12 hrs (x axis) and (B) 24 hrs. The same experimental paradigm was used for pathogenic mutant LRRK2 R1441C (C–D). n = 6 for all groups. Outer lines denote 1.5 fold expression differences. No genes expressed outside these levels reached significance (p<0.05). (E) Fibroblast expression profile of Control vs. pathogenic mutant LRRK2. WT (y axis, n = 10) and mutant LRRK2 G2019S (x axis, n = 8). Outer lines denote 1.5 fold expression differences. No genes expressed outside these levels reached significance (p<0.05) (F–H) Case control comparison of gene expression in brain tissue from G2019S vs iPD (F), G2019S vs control (G) and iPD vs control (H). n = 5 for G2019S and iPD, n = 15 for control. Outer lines denote 1.5 fold expression differences. No genes expressed outside these levels reached significance (p<0.05).

Having confirmed the expression of LRRK2 at the protein level in these systems, we carried out genome wide expression analysis. In the HEK cells we compared each cell line induced for 12 h or 24 h to its uninduced controls; no differences in gene expression were seen in either WT or R1441C expressing cells (Figure 2A–D). Similarly, no differences in gene expression were seen in fibroblast cell lines with heterozygous mutant (G2019S) LRRK2 compared to wild type controls (Figure 2E).

We also examined gene expression in brains of LRRK2 carriers compared to controls and to idiopathic PD (Figure 2F–H). For this series of experiments, we took tissue from the occipital cortex, where LRRK2 is expressed but where there is little evidence of the disease process as Lewy bodies are rarely formed here and there is no cell loss [18], [19]. Therefore, the aim of this analysis was to isolate the effects of LRRK2 from the disease process in the relevant tissue of human brain. Supporting the assumption that the occipital cortex is largely spared from the disease process, idiopathic PD cases showed no difference in gene expression compared to neurologically normal controls in this brain region (Figure 2H). However, as in the cell line analyses, there was no effect of LRRK2 mutation on gene expression compared to either neurologically normal controls or idiopathic PD cases (Figure 2F and G).

**Figure 3:**
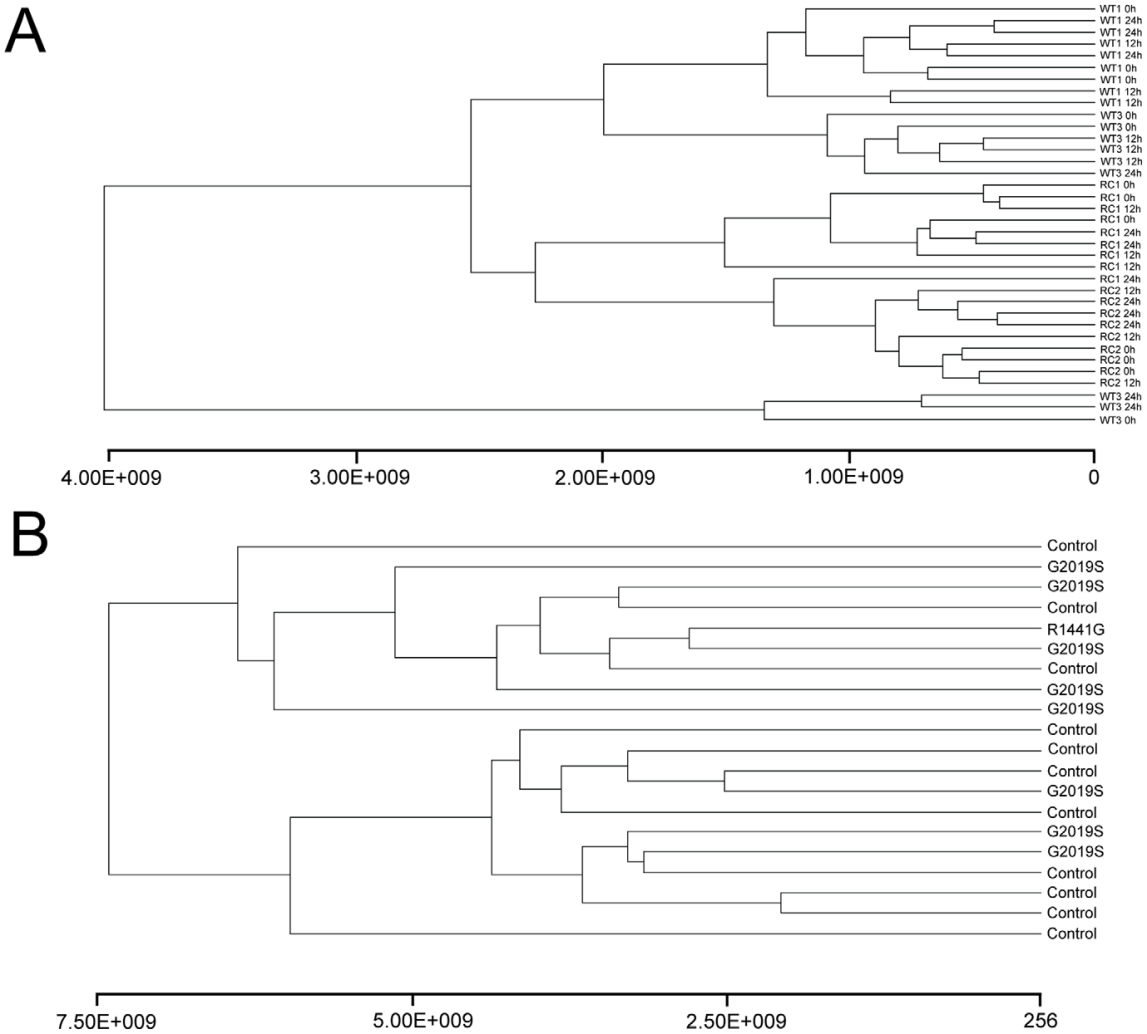
Gene expression dendrograms. (A) Unsupervised cluster analysis. Euclidian clustering of all induced LRRK2 time points (0, 12, 24 hrs) for WT and RC = R1441C. (B) The same clustering method used for the patient fibroblast expression data. CON = WT, G2019S and R1441G  =  pathogenic forms of LRRK2. In each case, separation of the groups was not possible.

We considered that simply looking for genes whose steady state expression might miss more subtle patterns in the data and therefore examined overall gene expression by unsupervised hierarchical clustering (Figure 3). Samples did not separate by genotype, again suggesting that there is no significant or consistent difference between the mutant and wild type cells.

## Discussion

This study assessed a series of patient fibroblast cells harbouring mutations in LRRK2, brain samples from G2019S carriers and a cell line system over-expressing LRRK2 wild type and mutant transgenes for alterations in gene expression due to the presence of mutant LRRK2. Overall, our data show that any differences in gene expression are lower than the relatively modest cutoffs (1.5 fold, p<0.05 after correction for multiple testing) used. In turn, this suggests that mutant LRRK2 does not elicit large changes in steady state mRNA levels within the cell under normal growth conditions. This is in contrast to a recent publication studying a series of mononuclear cells carrying the G2019S mutation [20] and data from *Drosophila* and a HEK cell model [8]. There are a number of possible reasons for the divergence in our data from those in these studies. With regard to the former, our cellular studies have been carried out on cultured cells in a monolayer, rather than primary cells in a suspension, and both fibroblasts and HEK cells represent a significantly different cell type to mononuclear cells. Focussing on the relevance of these systems to the biology of LRRK2, it is notable that both cell lines carrying plasmids allowing the inducible expression of LRRK2 and patient fibroblast samples have been used as model systems for examining the aberrant behaviour of mutant LRRK2 [8], [21]. The recent report of induced pluripotent stem cells (iPSCs) from a patient carrying a G2019S mutation offers a model system closer to the human brain for analysing gene expression alterations due to mutations in LRRK2 [22]. To date, however, it has not been possible to carry out gene expression analyses from multiple replicates of iPSC lines generated from multiple independent LRRK2 mutation carriers, a key requirement if changes in expression are to be detected and ascertained as significantly correlated with mutant LRRK2 alleles. To follow up on our analysis of LRRK2 mutations in model systems used to assess the biology of LRRK2, we examined the impact of mutant LRRK2 in a disease setting by carrying out a case control comparison of brain tissue from patients carrying mutations in LRRK2, idiopathic patient brain material and control samples derived from the Queen Square and Sun Health brain banks. Similar to our data from cellular analyses, no impact of mutations upon expression was observed once correction for multiple comparisons had been carried out. It should be noted that analysis of brain expression was limited by the small number of G2019S brain samples available for mRNA extraction, an issue that is also reflected in the number of pathological reports for LRRK2 cases [19].

Our results do not exclude the possibility that gene expression can be differentially altered by mutations in LRRK2 under conditions of stress, for example under starvation conditions, oxidative challenge or widespread cell loss [4]. Several recent papers have highlighted a potential role for LRRK2 in stress response *via* the mTOR pathway, and it is plausible that LRRK2 exerts an influence on gene expression following activation in response to cellular stress. With regard to alterations due to miRNA regulation, our data demonstrates no overall impact on mRNA levels. This does not exclude translational regulation as a mechanism through which a putative LRRK2 miRNA pathway could operate, although the recent publication by Guo *et al* emphasizes that miRNAs in mammalian cells predominantly function by directly impacting on mRNA levels rather than by premature translation termination or inhibiting ribosome formation or binding [9].

In summary, our data provides evidence from two independent cellular model systems and brain tissue from patients that LRRK2 mutations do not result in large alterations in basal gene expression under normal growth conditions. These data raise the possibility that mutant LRRK2 exerts its pathogenic affect through an alternative mechanism, for example through post translational modification *via* its kinase activity or through altered recruitment of other co-factors to an active enzymatic complex, aspects of the biology of LRRK2 that are undergoing investigation in a number of laboratories [23], [24].
