# Correction: Pathogenic LRRK2 Mutations Do Not Alter Gene Expression in Cell Model Systems or Human Brain Tissue

**DOI:** 10.1371/annotation/c12e4f9e-5aae-424b-a69f-fddf16976dc5

**Published:** 2012-01-27

**Authors:** Michael J. Devine, Alice Kaganovich, Mina Ryten, Adamantios Mamais, Daniah Trabzuni, Claudia Manzoni, Philip McGoldrick, Diane Chan, Allissa Dillman, Julia Zerle, Susannah Horan, Jan-Willem Taanman, John Hardy, Jose-Felix Marti-Masso, Daniel Healy, Anthony H. Schapira, Benjamin Wolozin, Rina Bandopadhyay, Mark R. Cookson, Marcel P. van der Brug, Patrick A. Lewis

There was a spelling error in the fifteenth author's name. The correct spelling is: Daniel Healy. 

